# Female fertility and infant survivorship increase following lethal intergroup aggression and territorial expansion in wild chimpanzees

**DOI:** 10.1073/pnas.2524502122

**Published:** 2025-11-17

**Authors:** Brian M. Wood, David P. Watts, Kevin E. Langergraber, John C. Mitani

**Affiliations:** ^a^Department of Anthropology, University of California, Los Angeles, CA 90095; ^b^Department of Human Behavior, Ecology and Culture, Max Planck Institute of Evolutionary Anthropology, Leipzig 04103, Germany; ^c^Department of Anthropology, Yale University, New Haven, CT 06511; ^d^School of Human Evolution and Social Change, Arizona State University, Tempe, AZ 85287; ^e^Institute of Human Origins, Arizona State University, Tempe, AZ 85287; ^f^Department of Anthropology, University of Michigan, Ann Arbor, MI 48109

**Keywords:** chimpanzees, lethal intergroup aggression, territorial expansion, female fertility, infant survivorship

## Abstract

Lethal coalitionary intergroup aggression is a conspicuous aspect of wild chimpanzee behavior. Evidence indicates that such violence can lead to territorial expansion, but whether this results in fitness benefits is unknown. Here, we show that female fertility and infant survivorship increased after males in the Ngogo chimpanzee community killed members of neighboring groups and expanded their territory. These findings demonstrate the fitness benefits of intergroup killing in one of our two closest living relatives and contribute to the debate regarding its adaptive significance.

Between 1998 and 2008, members of the Ngogo chimpanzee group in Kibale National Park, Uganda, killed 21 individuals from neighboring groups, including 13 victims living in a region to the northeast of their territory. After reducing the coalitionary strength of their neighbors, the Ngogo chimpanzees expanded their territory into an area previously inhabited by the victims. The expansion occurred in 2009 and included 6.4 square kilometers, representing a 22% increase in the size of their territory (1). These data indicate that chimpanzee lethal intergroup aggression results in access to additional territory and its associated food resources; however, the fitness consequences of such gains are unknown. To address this question, we compared female fertility and infant survivorship before and after the Ngogo community’s territorial expansion.

## Results

**Fig. 1.**
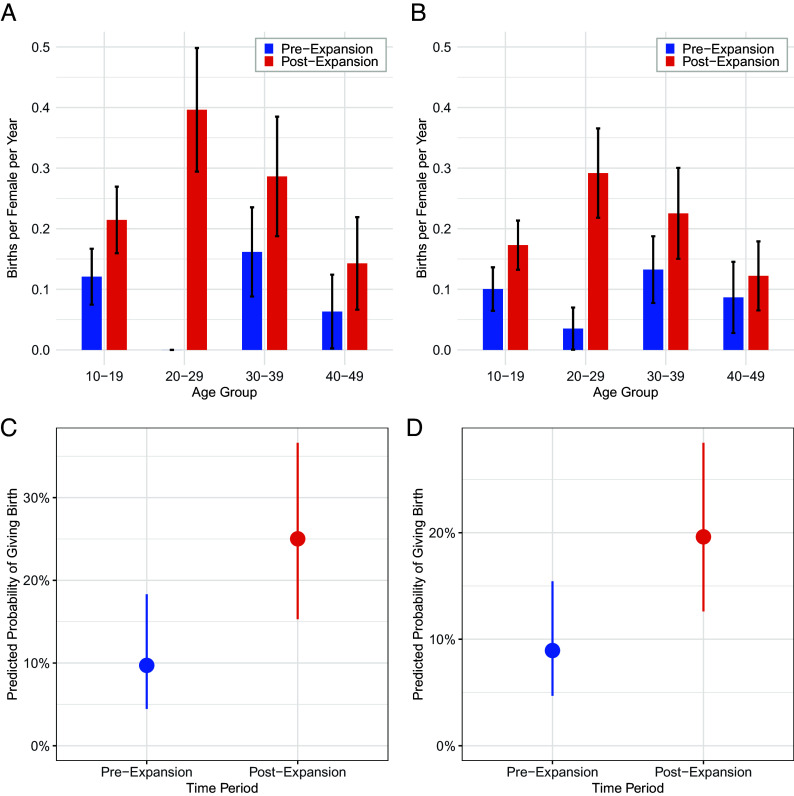
Fertility of Ngogo female chimpanzees before and after territorial expansion. (*A* and *B*) Age-specific fertility rates (ASFR) by 10-y age group in the 2-y (A) and 3-y (B) windows surrounding the expansion; error bars indicate ±1 SE. (*C* and *D*) Predicted annual probability of giving birth for females aged 10 to 49 from Bayesian hierarchical logistic regression models, for the 2-y (*C*) and 3-y (*D*) windows surrounding the expansion; points are posterior means and error bars show 95% credible intervals.

The number of recorded births at Ngogo more than doubled after territorial expansion. Females produced 15 infants in the three years preceding expansion compared with 37 in the three years following expansion. Because raw birth counts do not account for variation in female number or age structure, we also compared age-specific fertility rates (ASFR), which were higher after expansion. This pattern was consistent whether we analyzed two- or three-year periods before and after the event (Fig. 1 *A* and *B*).

Bayesian hierarchical models indicate that females were more than twice as likely to give birth after territorial expansion. This increase in birth rates was consistent across analyses comparing two-year periods [mean risk ratio = 2.75, 95% credible interval (CI): 1.4 to 5.2; Fig. 1*C*] and 3-y periods (mean risk ratio = 2.3, 95% CI: 1.3 to 4.0; Fig. 1*D*) around the territorial expansion.

**Fig. 2.**
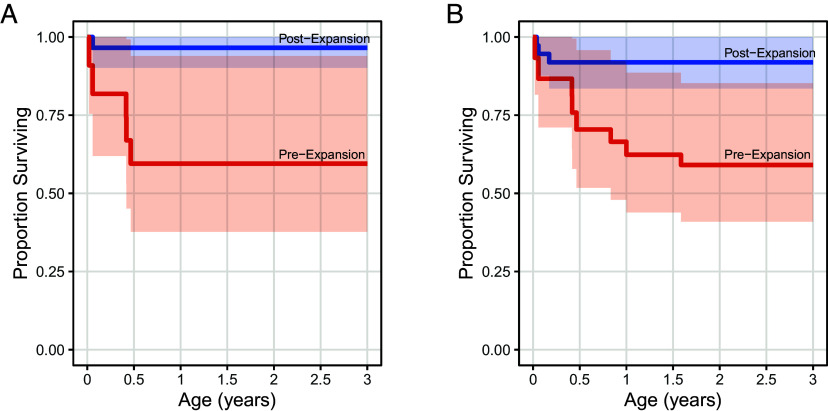
Kaplan–Meier survival curves showing the proportion of infants surviving to age three before and after expansion. Results compare two periods around expansion: (*A*) two-year windows and (*B*) three-year windows. Solid lines indicate the estimated survival functions, while ribbons represent 95% confidence intervals.

The increase in female fertility following territorial expansion coincided with marked improvements in offspring survivorship. In the three years before expansion, infants had a 41% probability of dying before age three, compared with just 8% after expansion. This difference reflects a 5.7-fold higher hazard of death in the pre-expansion period (HR = 5.7, 95% CI: 1.50 to 21.51; *P* = 0.011; Fig. 2*B*). A similar result emerged in analyses using two-year windows, with significantly lower survivorship pre-expansion (HR = 12.50, 95% CI: 1.47 to 108.1; *P* = 0.021; Fig. 2*A*).

In primates, infant survivorship influences female fertility because infant loss often leads to earlier resumption of ovarian cycling, reducing interbirth intervals (2). Because survivorship improved after the expansion, more frequent births in this period cannot be attributed to shortened interbirth intervals following infant deaths. Nor was it due to the presence of more reproductively active females without young infants in the postexpansion period (*SI Appendix*).

Improved infant survivorship amplified the fitness benefits of territorial expansion. For example, a female living to age 50 and reproducing under postexpansion conditions would be expected to produce 7.4 offspring surviving to at least age three, compared with only 2.2 under pre-expansion conditions (*SI Appendix*).

Chimpanzees eat fruit primarily, and we previously documented a secular increase in the fruit supply at Ngogo (3). Thus, increased food availability unrelated to territorial expansion may have contributed to the increase in female fertility. Visual inspection of phenology data presented in figures 3 and 4 of (3), however, suggests that fruit availability in the Ngogo chimpanzee territory remained stable or may have declined slightly following territorial expansion. Statistical comparison confirmed that the monthly proportion of fruiting trees in the three years after territorial expansion did not exceed the proportion of trees during the three-year pre-expansion period (Wilcoxon rank sum test: W = 827.5, *P* = 0.979, N = 36 each period).

## Discussion

Our results provide insights into the fitness consequences of lethal intergroup aggression in chimpanzees. Following multiple lethal attacks on members of neighboring groups, female chimpanzees at Ngogo gained access to additional territory and food resources. This likely improved their energetic condition, resulting in elevated fertility rates. These findings are consistent with prior research showing the strong influence of energetic condition on female primate fertility (4). The significant increase in territory size that accompanied the expansion also reduced within-group feeding competition. In primates, reduced feeding competition often improves female nutritional status and reproduction, including greater fertility (5). Higher infant survivorship, like the increase in female fertility, is a predicted outcome of the lethal intergroup aggression described here. Improved maternal energetic status could have contributed to the increase in infant survival. In addition, infanticide by members of other groups is a major source of infant mortality in chimpanzees (6). After killing many of their neighbors and reducing their collective strength, the Ngogo chimpanzees may have insulated their infants against this threat in the immediate aftermath of territorial expansion.

Despite extensive discussion of lethal coalitionary aggression as an evolved, adaptive strategy in chimpanzees, its fitness effects have not been investigated. One study of Gombe chimpanzees showed that female fertility was higher when the group’s territory size was larger but did not attribute changes in territory size to success in lethal intergroup aggression (7). Similarly, a study of Taï chimpanzees revealed that females had higher fertility in groups with more males, but did not show that more males in groups led to increased success in lethal intergroup aggression (8). Here, we demonstrate that a substantial increase in female fertility and offspring survival accompanied territorial expansion. Males, who carried out nearly all of the lethal intergroup aggression that enabled territorial expansion, shared these gains to female fitness because all Ngogo offspring were sired by group males (9). Although lethal intergroup aggression occurs across a wide array of taxa, territorial expansion following such events has been documented only rarely (10). Our findings provide direct evidence linking lethal coalitionary intergroup aggression to territorial expansion and enhanced fitness in chimpanzees.

Our results contribute to a long-standing debate regarding the significance of lethal intergroup aggression in chimpanzees. Despite considerable evidence consistent with the hypothesis that chimpanzee lethal intergroup violence is a normal part of their behavioral repertoire and that it has evolved by natural selection (6, 11), some argue that it is an artifact created by human impacts (12). The Ngogo chimpanzees feature prominently in this debate because they kill conspecifics in other groups frequently and occupy a relatively undisturbed forest with no human habitation bordering their territory. The observations presented here align squarely with the adaptive hypothesis.

In sum, territorial expansion following lethal intergroup aggression resulted in increased female fertility and decreased infant mortality in chimpanzees, one of our two closest living relatives. Whether similar processes played a role in human evolution remains an open question.

## Materials and Methods

We initiated fieldwork on the Ngogo chimpanzees in 1995 and have maintained continuous observations since that time (13). Throughout this study, a team of researchers and field assistants found and followed chimpanzees nearly every day and recorded births and deaths.

Methods for age estimation and demographic tracking are described in Wood et al. (14). While the Ngogo chimpanzees began using their newly acquired territory in June 2009, our definition of the postexpansion period includes an additional eight months, the length of gestation in wild chimpanzees, to account for the biological lag between the use of the new territory and observable changes in fertility. The postexpansion period thus begins on February 1, 2010. At the time of this study, the Ngogo chimpanzee community was extremely large, including 154 individuals at the beginning of the postexpansion period. Additional details regarding the study site, subjects, and data analysis can be found in SI Materials and Methods.

This study involved noninvasive behavioral observations of animals and was granted an exemption from review by the Institutional Animal Care and Use Committee at the University of Michigan.

## Supplementary Material

Appendix 01 (PDF)

## Data Availability

R code and data have been deposited in Open Science Foundation (15).
