# Supplementary material for: Female fertility and infant survivorship increase following lethal intergroup aggression and territorial expansion in wild chimpanzees
Source: Proc Natl Acad Sci U S A. 2025 Nov 17;122(47):e2524502122. doi: 10.1073/pnas.2524502122 (PMC12664014; doi:10.1073/pnas.2524502122)
Supplement: Supplementary file 1 — Appendix 01 (PDF) [file pnas.2524502122.sapp.pdf]

PNAS

Supporting Information for

Female fertility and infant survivorship increase following lethal intergroup aggression and territorial expansion in wild chimpanzees

Brian M. Wood[a,b]
David P. Watts[c]
Kevin E. Langergraber[d,e]
John C. Mitani[f]

[a]Department of Anthropology, University of California, Los Angeles; Los Angeles, CA 90095
[b]Department of Human Behavior, Ecology and Culture, Max Planck Institute of Evolutionary Anthropology; Leipzig, Germany 04103
[c]Department of Anthropology, Yale University; New Haven 06511
[d]School of Human Evolution and Social Change, Arizona State University, Tempe, AZ 85287
[e]Institute of Human Origins, Arizona State University, Tempe, AZ 85287
[f]Department of Anthropology, University of Michigan, Ann Arbor, MI 48109

Corresponding authors Brian M. Wood brianwood@anthro.ucla.edu or John C. Mitani mitani@umich.edu

This file includes:

Extended Materials and Methods
SI References

Materials and Methods

Study Site and Subjects. The Ngogo study site lies along the equator in the center of Kibale National Park, Uganda, at an altitude of about 1,400 m above sea level. The habitat is predominantly old-growth forest interspersed between colonizing forest regenerating from anthropogenic grasslands (*Pennisetum purpureum*) (1). The chimpanzees restrict their movements and activities largely to the forested areas and enter the remnant grasslands infrequently. The chimpanzees experience minimal human disturbance as their territory is surrounded by other chimpanzee groups. Consequently, they do not feed on human crops, nor have they ever been provisioned by us.

Prior research at Ngogo shows that 95% of female births occur between ages 14 and 47 (2). During the 36 months of the pre-expansion period, the community averaged 39.9 reproductively-aged females (SD = 1.4), compared to 50.3 (SD = 2.9) in the post-expansion period. Reproductively-active females, defined as those aged 14–47 without a nursing offspring younger than three years, accounted for 39.4% (SD = 11.6) of reproductively-aged females in the pre-expansion period and 36.6% (SD = 11.4) in the post-expansion period.

Choosing Comparison Periods Around the Territorial Expansion. To assess fertility and infant survivorship changes associated with the territorial expansion, we must define appropriate observation windows on either side of the event. The choice of window length involves trade-offs. Shorter windows are preferable for causal inference, as they reduce exposure to unmeasured confounders and align with the expectation that any benefits of territorial expansion are transient. However, shorter windows yield fewer observations, increasing sampling uncertainty and reducing statistical precision. Longer windows provide more observations and greater statistical power, but risk diluting the effects of territorial expansion and conflating them with other time-dependent processes. In our analyses, we opted to present both two- and three-year observation windows before and after the expansion. Our models allow us to quantify and visualize the sampling uncertainty associated with the choice of window length. In addition to fertility and infant

survivorship, interbirth intervals are a component of female reproductive success that have been investigated in studies of primate female reproduction. However, because successful, closed birth intervals at Ngogo average 5.5 years (3) and exceed the two- and three-year periods we considered around territorial expansion, we did not examine this variable in our analyses.

Bayesian Hierarchical Modeling of Fertility. To model fertility (births per female per year) in the pre-expansion and post-expansion periods (figure 1), we employed a Bayesian Generalized Linear Mixed Model (GLMM) implemented in R using the *brms* package (4). The outcome variable is whether an Ngogo female gave birth or not in a given observation year. The fixed effect predictor variables were 1) the age of the female (categorical, 10 year age groups), and 2) the time period (categorical, pre-expansion period; post-expansion period). Random intercepts were used to model repeated measures of the same females. For this analysis, we only considered those age groups where at least one birth occurred, i.e. 10-19, 20-29, 30-39, and 40-49.

For these analyses, we defined years of observation not based on the Gregorian calendar year but based on the timing of the territorial expansion. The post-expansion period is defined as beginning on February 1, 2010, and we calculate our one-year periods of observation in reference to that date. For example, employing a three-year observation window entails comparing the fertility of females in the three pre-expansion years (February 1, 2007 - January 31, 2010) to three post-expansion years (February 1, 2010 - January 31, 2013).

Model 1 facilitates a comparison of two years before expansion to two years after expansion. This dataset contains 222 one-year observations from 65 individual females, and records 41 births. The variable *age_group* categorizes individuals into four decade-based age groups, based on their age at the start of the observation year: 10–19, 20–29, 30–39, and 40–49 years. In the data used to fit model 1, the pre-expansion period includes 103 observations of females, and the post-expansion period includes 119 observations.

Model 2 facilitates a comparison of three years before expansion to three years after expansion. The dataset used for fitting this model includes 336 one-year observations from 74 individual females during the pre-expansion period (153 observations) and the post-expansion period (183 observations). In the dataset used to fit model 2, 52 births were recorded.

The R model formula code for fitting models 1 and 2 using *brms* is as follows:

```
gave_birth ~ time_period + age_group + (1 | female)
```

Weakly informative Student's t(3, 0, 2.5) priors were applied to the model intercept and group-level standard deviations. All fixed effects, including those for *time_period* and *age_group*, were assigned flat (non-informative) priors. We assessed model convergence by examining trace plots, which showed that the four MCMC chains overlapped well and mixed thoroughly. All parameters had R-hat values of 1.00 and effective sample sizes exceeding 10,000, indicating reliable estimation of credible intervals.

We used the *conditional_effects* function from the *brms* R package to estimate the predicted probability of giving birth during pre- and post-expansion periods, illustrated in figure 1C and figure 1D. These estimates represent expected probabilities averaged across individual random effects and age group fixed effects, and include 95% credible intervals.

Kaplan-Meier Infant Survivorship Analysis. We estimated infant survivorship during the pre-expansion and post-expansion periods, using both two-year and three-year windows for each period. Individuals included in the analysis were all those who were less than three years old at any time during either period. Kaplan–Meier survival curves to age three were estimated using the *survfit* function in the R package *survival* (5), and differences between periods were assessed with a Cox proportional hazards model (6). We tested the proportional hazards assumption with the *cox.zph* function. For the three-year windows, the test for the period variable yielded $\chi^2 = 1.89$ with 1 degree of freedom ($P = 0.17$), providing no evidence of violation of the proportional hazards assumption. Results were similar for the two-year windows ($\chi^2 = 0.52$, df = 1, $P = 0.47$).

Calculating Female Fitness in Pre- and Post-Expansion Periods. To illustrate the potential fitness consequences of territorial expansion, including changes in both female fertility and infant survivorship, we modeled the number of offspring surviving to at least age three that a female would be expected to produce under pre- and post-expansion conditions. In this analysis, we simulated a female living from age 10 to 49 who experienced the age-specific fertility rates (ASFR) estimated from model 2 during the three years before or after expansion, and whose infants experienced the mean survivorship to age three for the corresponding period, estimated from the Kapan-Meier survivorship analysis.
Fertility values for each 10-year age class were:

- Pre-expansion: 10–19: 0.0925; 20–29: 0.108; 30–39: 0.116; 40–49: 0.0641
- Post-expansion: 10–19: 0.198; 20–29: 0.225; 30–39: 0.242; 40–49: 0.141

In the analysis of offspring survivorship using three-year time windows (figure 2B), survivorship to age three, S(3), was 0.667 in the pre-expansion period and 0.919 in the post-expansion period. For each age group, the expected number of infants surviving to age three was calculated as 10 × ASFR × S(3). Summing across all age groups provided the estimated lifetime number of offspring surviving to age three in each period.

SI References

1. J. S. Lwanga, Forest succession in Kibale National Park, Uganda: implications for forest restoration and management. *Afr. J. Ecol.* **41**, 9–22 (2003).
2. B. M. Wood, *et al.*, Demographic and hormonal evidence for menopause in wild chimpanzees. *Science* **382**, eadd5473 (2023).
3. M. Emery Thompson, Comparative reproductive energetics of human and nonhuman primates. *Annu. Rev. Anthropol.* **42**, 287–304 (2013).
4. P.-C. Bürkner, Advanced Bayesian multilevel modeling with the R package brms. *RJ.* **10**, 395–411 (2018).
5. T. M. Therneau, A package for survival analysis in R. https://cran.r-project.org/web/packages/survival/vignettes/survival.pdf. Deposited 17 December 2024.
6. T. M. Therneau, P. M. Grambsch, *Modeling Survival Data: Extending the Cox Model* (Springer, 2000).
